# Implementation of digital remote postoperative monitoring in routine practice: a qualitative study of barriers and facilitators

**DOI:** 10.1186/s12911-024-02670-5

**Published:** 2024-10-21

**Authors:** Kenneth A. McLean, Alessandro Sgrò, Leo R. Brown, Louis F. Buijs, Kirsty Mozolowski, Luke Daines, Kathrin Cresswell, Mark A. Potter, Matt-Mouley Bouamrane, Ewen M. Harrison

**Affiliations:** 1 Department of Clinical Surgery, University of Edinburgh, 51 Little France Crescent, Edinburgh, EH16 4SA UK; 2 Centre for Medical Informatics, Usher Institute, University of Edinburgh, Edinburgh, EH16 4UX UK; 3 Colorectal Unit, Western General Hospital, Edinburgh, EH4 2XU UK; 4 Division of Computing Science, Faculty of Natural Sciences, University of Stirling, Stirling, UK

**Keywords:** Qualitative, Implementation, Digital health, Surgery, Surveillance

## Abstract

**Introduction:**

Remote monitoring can strengthen postoperative care in the community and minimise the burden of complications. However, implementation requires a clear understanding of how to sustainably integrate such complex interventions into existing care pathways. This study aimed to explore perceptions of potential facilitators and barriers to the implementation of digital remote postoperative monitoring from key stakeholders and derive recommendations for an implementable service.

**Methods:**

A qualitative implementation study was conducted of digital remote postoperative wound monitoring across two UK tertiary care hospitals. All enrolled patients undergoing general surgery, and all staff involved in postoperative care were eligible. Criterion-based purposeful sampling was used to select stakeholders for semi-structured interviews on their perspectives and experiences of digital remote postoperative monitoring. A theory-informed deductive-inductive qualitative analysis was conducted; drawing on normalisation process theory (NPT) to determine facilitators for and barriers to implementation within routine care.

**Results:**

There were 28 semi-structured interviews conducted with patients (*n* = 14) and healthcare professionals (*n* = 14). Remote postoperative monitoring was perceived to fulfil an unmet need in facilitating the diagnosis and treatment of postoperative complications. Participants perceived clear benefit to both the delivery of health services, and patient outcomes and experience, but some were concerned that this may not be equally shared due to potential issues with accessibility. The COVID-19 pandemic demonstrated telemedicine services are feasible to deliver and acceptable to participants, with examples of nurse-led remote postoperative monitoring currently supported within local care pathways. However, there was a discrepancy between patients’ expectations regarding digital health to provide more personalised care, and the capacity of healthcare staff to deliver on these. Without further investment into IT infrastructure and allocation of staff, healthcare staff felt remote postoperative monitoring should be prioritised only for patients at the highest risk of complications.

**Conclusion:**

The COVID-19 pandemic has sparked the digital transformation of international health systems, yet the potential of digital health interventions has yet to be realised. The benefits to stakeholders are clear, and if health systems seek to meet governmental policy and patient expectations, there needs to be greater organisational strategy and investment to ensure appropriate deployment and adoption into routine care.

**Trial registration:**

NCT05069103.

**Supplementary Information:**

The online version contains supplementary material available at 10.1186/s12911-024-02670-5.

## Introduction

The early postoperative period is often associated with high patient morbidity, posing a significant burden to patients and health systems [1]. However, patients are increasingly discharged earlier in their postoperative course [2]. While early discharge can provide clear benefits to patients and health systems in terms of cost and recovery, the trade-off is that up to 40% of postoperative complications now occur in community settings [3]. This places addition burden on patients and community services to recognise and respond appropriately to potential postoperative complications, which can lead to patients feeling isolated, distressed, and unsupported in the community [4, 5]. Furthermore, at a time where many healthcare systems are overstretched, there is evidence that utilisation and efficiency of postoperative care can be improved though reducing unnecessary healthcare attendance [6]. In recognition of these issues and the increasing opportunities afforded by the accessibility of mobile and wireless technologies [7, 8], the development of digital health interventions (DHIs) for remote. In recognition of these issues and the increasing opportunities afforded by the accessibility of mobile and wireless technologies [6, 7], the development of digital health interventions (DHIs) for remote postoperative monitoring has accelerated in recent years to facilitate care and rapid response to complications in the community [8]. However, few studies have evaluated the implementation of these postoperative interventions in practice, and there is widespread acknowledgement that the potential of DHIs have yet to be realised within healthcare systems [7].

Implementation of a complex health intervention such as remote postoperative monitoring can be particularly challenging given this inherently involves disruption to existing care pathways [9]. The field of implementation science emphasises how qualitative studies are crucial to provide a comprehensive and nuanced understanding of these issues to inform the successful integration of these interventions into routine care [10]. However, few of these have been conducted to date on this topic [11–13]. Therefore, this qualitative study aimed to identify potential facilitators and barriers for the implementation of digital remote postoperative monitoring from the perspectives of key stakeholders and derive recommendations for an implementable service in a national health service context such as the UK National Health Service (NHS).

## Methods

### Research context

“*ImplementatioN of Remote surgical wOund Assessment during the coviD-19 pandEmic*” (INROADE) was a single-arm implementational study across two tertiary hospitals in a large UK health board (NHS Lothian). Based on their own degree of concern regarding potential complications, patients have access to telephone-based triage (“*NHS 111*”) by trained staff, or their general practitioner (GP) or emergency department for clinical review. Within local postoperative care pathways, patients at high-risk for complications have access to nurse-led telephone triage to provide advice on postoperative recovery.

The principal components of the DHI explored within INROADE have been previously reported [14] (Supplementary Fig. 1). Enrolled patients had access to an online tool throughout the early postoperative period (day 1 to 30). This included the ability to submit images of their surgical wound(s), patient-reported symptoms related to surgical-site infection (SSI), and optional free text for additional context. This submission was triaged by a qualified clinician as either: (1) no clear evidence of SSI (low-risk), but with recommendation to attend healthcare services if ongoing concerns; (2) possible evidence of SSI (medium-risk), with recommendation to attend their GP for clinical review; (3) probable evidence of SSI (high-risk), with recommendation to attend emergency services for clinical review.

### Study design

This qualitative study was nested within INROADE to evaluate stakeholder (healthcare staff and patient) views in relation to the feasibility, acceptability and readiness of telemedicine in postoperative care, with the intervention as an exemplar. It is reported according to “*Consolidated criteria for reporting qualitative research*” guidelines [15]. All INROADE patients were eligible for interview – this included adults (≥ 16 years) who underwent abdominal general surgery (at least one surgical incision into the peritoneal cavity or gastrointestinal tract). Key inclusion criteria were smartphone ownership (with internet access) and capacity to provide informed consent [14]. Patients could volunteer to be interviewed after 30-day follow-up. Similarly, all clinical and nursing staff involved in postoperative care were eligible. Their involvement was respondent-led, with gatekeepers in primary and secondary care providing information regarding the opportunity for involvement. Criterion-based purposeful sampling based on interviewee characteristics [16] was used to gain a wide range of perspectives. For patients, this included age (< 65 years, ≥ 65 years), operative urgency (elective, emergency), surgical speciality (upper gastrointestinal or colorectal), socioeconomic status (index of multiple deprivation decile [17]), and SSI occurrence (yes, no). For staff, this included clinical role (doctor, nurse), stage of training (junior doctor, consultant), and location (community or hospital).

### Data collection

Semi-structured interviews were conducted, with separate interview guides for patients and staff (**Appendices A-B**). These explored the implementation of remote postoperative monitoring within the health service, with SSI as an exemplar use-case to facilitate discussion. This was informed by Normalisation Process Theory (NPT), a widely used theoretical framework in implementation science used to understand how stakeholders adopt complex health interventions interventions – including digital health interventions - and consider them “normalised” practice [18]. This encompasses four domains (Coherence, Cognitive Participation, Collective Action, Reflexive Monitoring), with associated subdomains [19, 20] (Supplementary Table 1). The interviews were conducted by one interviewer, a trainee surgeon involved in patient recruitment (KAM) which each interview lasting 30–60 min. Due to the COVID-19 pandemic, all interviews were conducted remotely, with consent for recording. All interviews were transcribed verbatim by independent medical transcribers, with the text anonymised prior to being returned to the research team for comment and/or correction. Participants were accrued until data saturation was achieved [21]. This was approved by the West of Scotland Research Ethics Committee (Number: 21/WS/0046).

### Data analysis

An inductive/deductive hybrid thematic analytic approach was adopted to balance the theoretical rigor provided by themes derived from an existing framework, while recognising the complex nature of DHIs may benefit from an inductive approach to identify emerging themes [22]. Firstly, NPT was used as the deductive coding framework to which initial findings were mapped [19] (Supplementary Table 1). This is frequently used as a qualitative framework within similar studies in telehealth [23]. Secondly, within each NPT subdomain, emerging subthemes were inductively coded and the sentiment recorded using a SWOT (Strengths, Weaknesses, Opportunities, Threats) approach [24]: facilitators (strengths, opportunities), barriers (threats, weaknesses), and mixed (interrelated facilitators and barriers). Each transcript was initially coded by one author (KAM), with a sample double coded by another author (MB). Both coders were academic researchers and/or clinicians with experience in qualitative research methods, and any disagreement was discussed and resolved with a third member (EMH). Thirdly, the NPT subdomains were reordered to address specific research questions regarding stakeholder perceptions of remote postoperative monitoring: (1) Is the intervention an appropriate solution for the clinical problem; (ii). Is the intervention of clinical benefit; (iii). Is the intervention one stakeholders would be willing to engage with; and (iv). What organisational support would be required to deliver the intervention? Fourthly, triangulation was performed between the different stakeholder groups and subgroups to explore any distinct experiences and viewpoints [25]. Finally, participants interviewed were also asked the minimum sensitivity and specificity they would consider acceptable for remote triage for a postoperative complication like SSI. The interquartile ranges (IQR) were used to determine “*minimally acceptable criteria*” (MAC) for patients and healthcare staff [26]. These were triangulated with qualitative findings as part of a mixed-methods sub-analysis [25, 27]. All interviewees were assigned anonymised numbers according to their role which was used to identify the source of illustrative quotations - patients (“P”), surgeons (“S”), GPs (“GP”), and nurses (“N”). Analysis was facilitated using QRS Nvivo ^®^ Version 12.

## Results

### Interviewee overview

All 200 patients enrolled in the INROADE study between the 1st July 2021 and 30th April 2022 were invited to interview, with 40 (20.0%) agreeing and 14 who participated. Only patients of White ethnicity were able to be interviewed, and were more likely to be female sex (78.6%) (Supplementary Table 2). Furthermore, 14 healthcare professionals involved in postoperative care were invited for interview. These consisted of hospital staff (3 consultant surgeons, 3 trainee surgeons, and 3 advanced nurse practitioners) and GPs (*n* = 5). The median length of service across all participating staff was 11.5 years (IQR: 7.2 to 19.0).

**Fig. 1:**
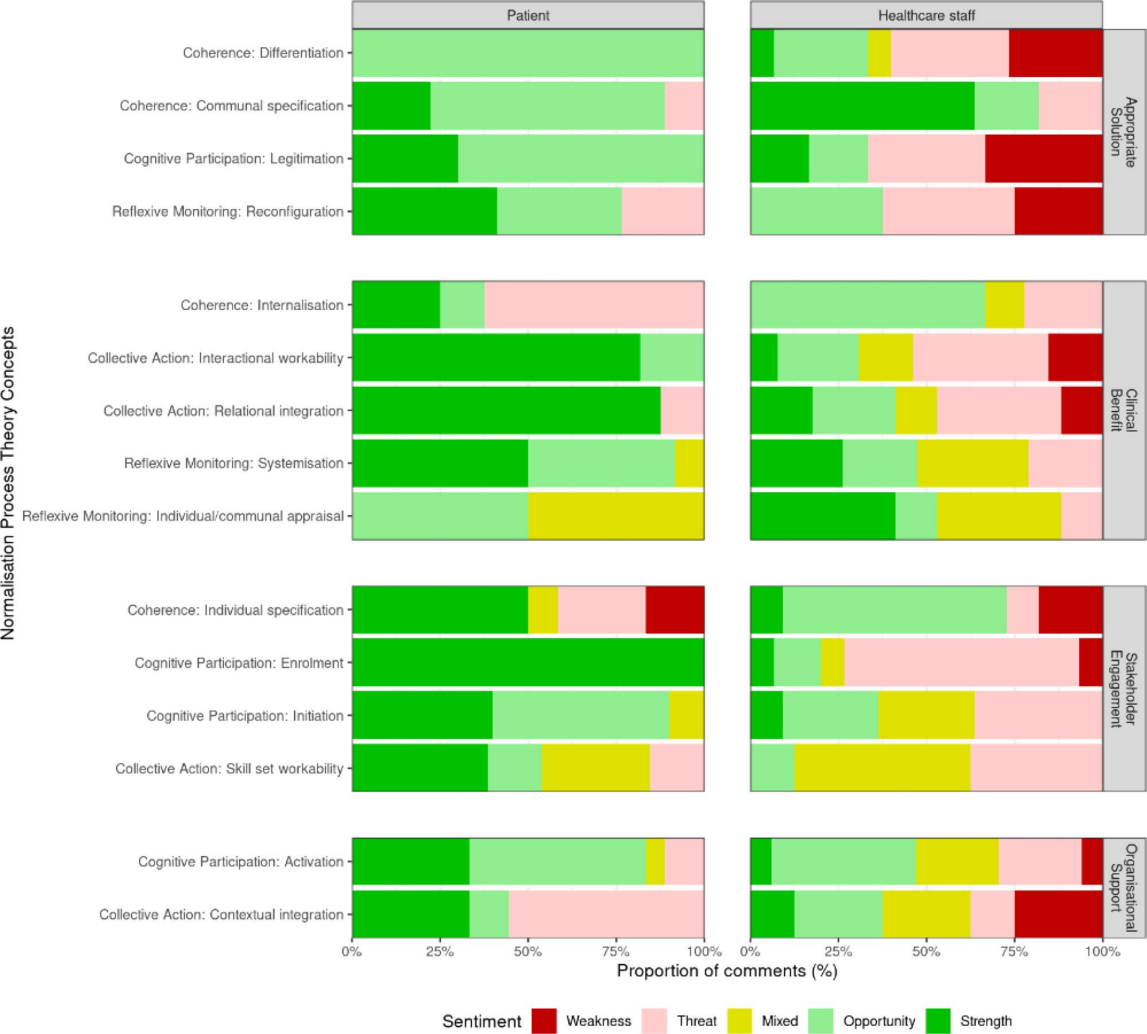
Comparison of stakeholder sentiment regarding implementation of remote postoperative monitoring, according to Normalisation Process Theory concepts

**Fig. 2:**
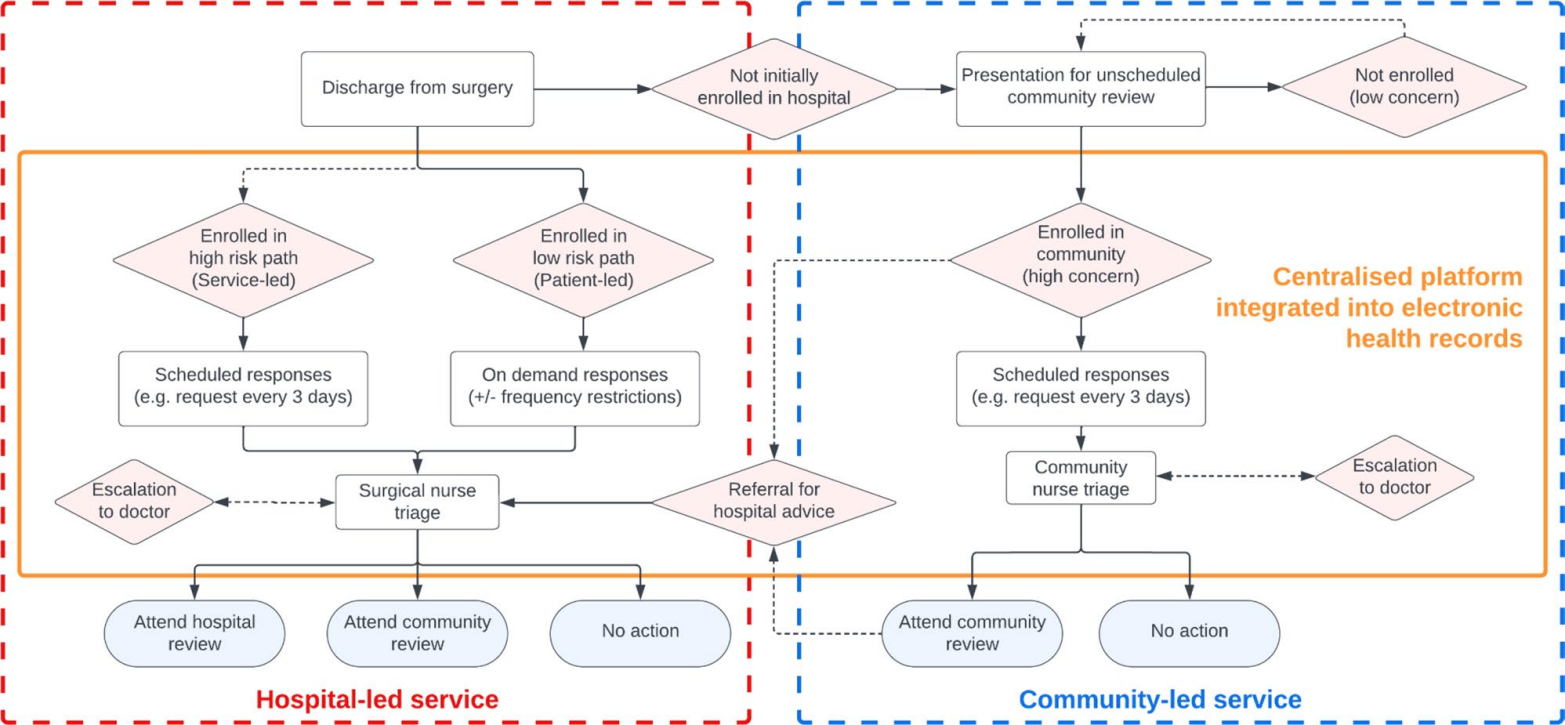
Stakeholder-derived pathway for a remote postoperative monitoring service within routine care. The proposed pathway is composed of hospital-led and community-led service components, with a central platform integrated into both electronic health records to promote shared care. The pathway starts with the patient being discharged from hospital, with rectangle boxes represent processes, diamond boxes (pink) represent decisions, and oval boxes (blue) represent terminators. Optional pathways are represented with dotted lines

Patients interviewed were generally more likely to view remote postoperative monitoring positively compared to healthcare staff who could envision both facilitators and barriers to implementation (Fig. 1). This was consistent at the individual level for patients and staff, regardless of their respective roles (Supplementary Figs. 2–3). The key barriers and facilitators to the implementation of remote postoperative monitoring were summarised (Table 1), and reported in detail below. Findings were synthesised into a remote postoperative monitoring pathway with a focus on normalisation within routine care (Fig. 2).

### Appropriate solution for the clinical problem

1. Facilitator: Remote postoperative monitoring fulfils an unmet need.

An important step towards a novel intervention becoming normalised, consists in an agreement emerging among stakeholders that there is an appropriate clinical problem (i.e. an unmet care or service need) that the intervention has an important role in solving and that it differs in doing this from already existing and established practices [18]. From a patient perspective, there was a strong consensus that the advice received on discharge was either not given or basic (*“I don’t remember anything written*,* verbally it was it looks fine…I think I was told any worries to phone back or speak to GP. Basic advice”* [R3]) and this was reinforced by the experiences of community healthcare staff (*“Sometimes it is maybe a slight communication problem [between patients and the surgical team]. And that is difficult sometimes to resolve because you don’t know if it’s just them not picking things up or whether it wasn’t made clear.”* [GP4]). Despite this, patients generally expressed a reluctance to “bother” healthcare workers with questions (*“you are thinking like I’ve already been in for six days and I’ve taken up enough time and I don’t really want to take up anymore of peoples time”* [R6]), however some felt there were insufficient alternate information resources to access post-discharge in case of concerns (*“I can Google one site and get one information*,* then I Google another and it completely contradicts.”* [R4]).

**Table 1:** Summary of barriers and facilitators identified regarding implementation of remote postoperative monitoring, according to normalisation process theory concepts

Domain	Barriers and facilitators to implementation of remote postoperative monitoring
Appropriate solution	1. Facilitator: Remote postoperative monitoring fulfils an unmet need, including in other surgical specialties (Cognitive Participation – Legitimation; Reflexive Monitoring – Reconfiguration, Coherence - Communal specification)
	2. Facilitator: There is differentiation from routine practice: both existing telemedicine services and in-person assessment (Coherence - Differentiation)
Clinical Benefit	1. Mixed facilitator and barrier: There is a clear clinical benefit, but concern that this may not be shared for all patients (Coherence – Internalisation; Collective Action - Interactional workability & Relational integration).
	2. Mixed facilitator and barrier: There is consensus on how the overall effectiveness of remote postoperative monitoring should be determined. However, there are conflicting prioritisation between patients and healthcare staff (Reflexive Monitoring – Systemisation & Individual/communal appraisal).
Stakeholder engagement	1. Facilitator: Stakeholders are generally willing to participate in remote postoperative monitoring, with online communication having been normalised over the pandemic (Cognitive Participation - Initiation).
	2. Mixed facilitator and barrier: Patients are motivated to participate and generally feel comfortable with the tasks required, although some may struggle without additional training or support at home (Coherence - Individual specification; Cognitive Participation – Enrolment & Initiation; Collective Action - Skill set workability).
	3. Facilitator: Healthcare staff feel they can perform remote triage using information from postoperative monitoring, with a clear consensus that this only required sufficient experience and was not limited to only doctors (Coherence - Individual specification; Cognitive Participation – Enrolment & Initiation; Collective Action - Skill set workability).
Organisational support	1. Facilitator – The COVID-19 pandemic normalised telemedicine services, with examples of nurse-led remote postoperative monitoring are currently supported within local care pathways (Collective Action - Contextual integration).
	2. Mixed facilitator and barrier – Integration with existing health information infrastructures (Collective Action - Contextual integration).
	3. Barrier – Healthcare staff are overstretched, and so additional and specific staff time would need to be allocated (Collective Action - Contextual integration).
	4. Barrier – There is a discrepancy between patient expectations regarding digital health and the capacity of healthcare staff to deliver. Healthcare staff preferred patient-led follow-up and while patients preferred service-led follow-up. Due to limited capacity at present, healthcare staff feel resources should be prioritised to those at highest risk, not all patients (Cognitive Participation – Activation; Collective Action - Contextual integration).

Healthcare staff generally felt that postoperative complications like SSI were among the most common issues encountered in surgical patients across the community and hospital settings, although remained a relatively small proportion of the overall workload. However, complications requiring referral to hospital were felt to be rarely encountered on an individual level in the community (*“Often people are worried about how the wound is looking*,* often because they’re a bit red or there’s bruising around it. Or there is a bit of pain*,* something like that”* [GP1]), and even among those who attended hospital, few needed readmission (*“The vast majority just need antibiotics and reassurance - generally we do not get involved”* [S6]). This is aligned with quantitative findings regarding readmission with SSI in INROADE [14]. Furthermore, it was noted that this need was not just limited to general surgery patients (*“Obstetrics*,* actually - they’re probably one of the higher ones that we see not infrequently…they seem to be home really quickly*,* but they are ones that do get sick sometimes”* [GP4]).

Overall, there was clear agreement among participants that a key purpose of remote postoperative monitoring would be to facilitate the early diagnosis of postoperative complications. Furthermore, there was a clear desire from all staff for enhanced communication between primary and secondary care. In cases of diagnostic uncertainty in the community, GPs perceived limited ability to seek further information or advice from hospital teams (*“you probably would just make a judgement yourself rather than try to get a hold of the surgical team because it would just take too long.”* [GP1]). This was mirrored by hospital staff who were often unclear on prior management in the community when patients attended for review (*“They might have actually seen the district nurse*,* but we maybe don’t know about it”* [S3]). Furthermore, while several GPs interviewed felt confident in management of postoperative complications, there was general acknowledgement the ability to more easily monitor wounds and request advice remotely from hospital teams would increase confidence in determining when antibiotic prescription is not required and reduce inter-practitioner differences in management (*“Having the ability to ask the expert to get advice without needing to send that patient in…any time there is the ability to ask for specialist advice then as the GP you can act on is a good thing.”* [GP4]).

2. Facilitator: Remote postoperative monitoring demonstrates differentiation from routine practice.

Normalisation also requires it to be clear to participants how the intervention is distinct from existing approaches and what are the additional benefits of the new approach [18]. Remote telephone and teleconference consultations have been routinely used across the NHS since the pandemic [28, 29]. However, in our study, the capability for digital services to obtain high-resolution visual data for wound review was perceived by healthcare staff as a distinct advantage offered in the context of the exemplar intervention (*“You know*,* obviously the more information you get*,* the better. A telephone call isn’t as good as a photograph”* [N2]). There was also differentiation from in-person clinical assessment, although this was regarding the limitations posed to the diagnostic process (*“When I see wound infections I would even without speaking to them ask them to come in*,* because I do not think you can do a full assessment.”* [GP2]).

### Clinical benefit

1. Overall mixed facilitator and barrier: There is a clear clinical benefit, but concern that this may not be shared for all patients.

A fundamental part of convincing participants to integrate a new intervention within their practice is that there will be a positive impact on the delivery of healthcare. There was agreement among participants that remote postoperative monitoring can achieve the intended purpose of allowing faster identification and potentially more reliable diagnosis of complications like surgical-site infection. As such, there was a shared expectation that this would reduce the severity of SSI and potential sequelae (*“The earlier you can catch something*,* the earlier you can deal with it*,* which is always going to be better for the patient”* [N1]). Furthermore, there was felt to be clear benefits to health service utilisation, with patients and staff generally more comfortable with earlier discharge home with this as a safety-net to provide reassurance (*“I think if you could monitor patients more closely but from home*,* certainly [you would] have more confidence sending patients home”* [S4]; *“We don’t want to go back to the old days where you didn’t get out of hospital until you were a 100%”* [P6]), and that unnecessary healthcare attendance in the community and hospital would be avoidable if implemented (*“So if we were able to cut a few off at the pass there*,* that would save them a lot of toing and froing for the patient*,* a lot of unnecessary waiting around.”* [N2]; *“And that was really good*,* I mean because again it just saved so much hassle of having to contact the GP”* [P5]).

From a patient perspective, the early post-discharge period was seen as isolating and uncertain (*“You knew someone was having a look at that*,* so you did not feel like you were on your own*,* because you really were on your own.”* [P13]). Both staff and patients perceived a benefit in allowing patients to be more empowered within their own healthcare, while providing valuable reassurance regarding potential wound concerns (*“What I liked about it is that I felt like it kind of trained me to recognise issues because this form had all these questions*,* and I don’t think I knew [beforehand].”* [P8]). Overall, this was felt to improve the perceived quality of care they received and their overall postoperative experience (*“It’s nice to know someone cares that much and they really want to make sure you are okay and how things are going.”* [P14]).

However, it was also noted that these benefits may not be shared equally and that the requirement for digital access and literacy could form a barrier to participation (*“Some people in whom the technology*,* unless were funding it*,* might not be available to them”* [S2]). Patients who were elderly or non-native language speakers were highlighted as particular subgroups who could be negatively affected. Conversely, remote postoperative monitoring was expected to improve access for some subgroups who may struggle to engage with existing services, for example, patients in remote settings or those with mobility or hearing impairments.

2. Overall mixed facilitator and barrier: There is consensus on how the overall effectiveness of remote postoperative monitoring should be determined. However, there are conflicting prioritisation between patients and healthcare staff.

Normalisation also requires an understanding of how participants will judge the overall effectiveness of remote postoperative monitoring. There was consensus among participants that at a health system level, remote postoperative monitoring needs to demonstrate a clear cost-benefit to the health system and/or demonstrable improvement of clinical outcomes (*“The only way to justify…is if there is demonstrably proven to be better for patient outcomes and health economics”* [S2]; *“Somebody obviously at the other end is having to spend time reviewing that when normally I guess normally they wouldn’t necessarily be…if it can save the resources without other things happening*,* it [would be] fantastic.”* [P5]).

**Fig. 3:**
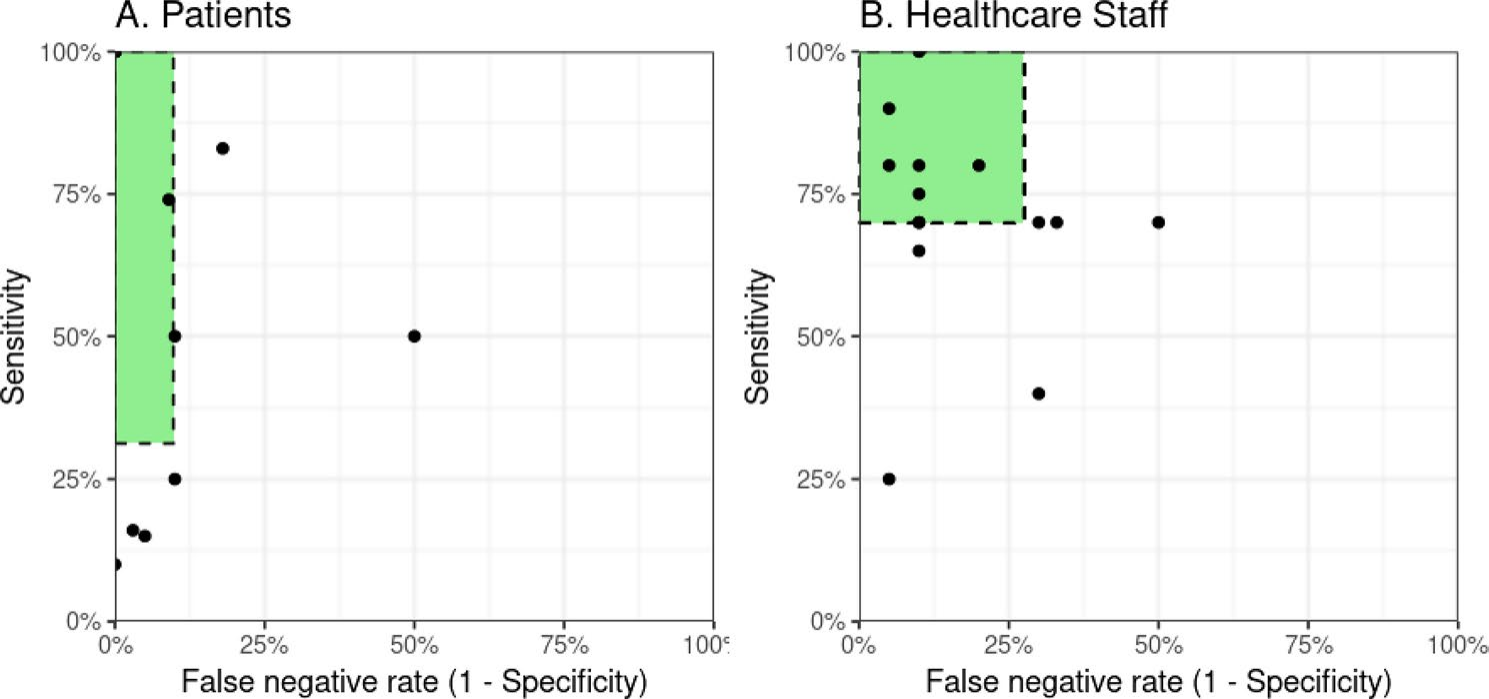
*Minimally acceptable criteria (MAC) for remote triage of surgical-site infection.* The green box represents the sensitivity and specificity for remote triage of surgical-site infection which would be considered acceptable to the majority of participants interviewed. The thresholds are based on the lower quartile (Q1) of responses

Furthermore, in the context of screening methods, it is important that triage accuracy based on remote review meets the sensitivity and specificity expected by participants (Fig. 3). There was no clear consensus among individual participants, however, healthcare staff generally had higher expectations for sensitivity (70.0%, IQR: 70.0–80.0%) compared to patients (78.5%, IQR: 31.2–100.0%). This was predominately driven by concerns among healthcare staff regarding overburdening health service (*“If we were bringing in four patients a day. And none of them had wound infections. Within a week*,* it would be shut down…I think as a department our tolerance would be 0.”* [N2]), and causing unnecessary distress and inconvenience to patients for false positives (*“you don’t want patients panicking because something’s triggered.”* [S3]). In contrast, patients sought to prioritise the minimisation of false negatives (specificity = 98.5%, IQR: 90.2–100.0%), whereas healthcare staff would tolerate a higher number (specificity = 90.0%, IQR: 72.5–90.0%), so long as these were not severe infections and they were subsequently identified as time progressed (Fig. 3). They highlighted that patients already receive worsening advice in areas of diagnostic uncertainty as part of routine care and that remote monitoring provides a further safety net by allowing enhanced monitoring over time (*“this is our day-to-day practice*,* this is how we function in general practice. So in a way that we would see that wouldn’t be it’s an adverse event”* [GP5]; *“If it had become infected I could not really blame you*,* as you were telling me it’s okay*,* but always err on the side of caution. It was not taking away all the responsibility from me. I would think it would be my fault if I did not do anything. You are not in the room*,* so it is the best you can do*,* but it does not take away personal responsibility.”* [P11]). Nonetheless, the concern regarding the risk posed by false reassurance did persist for some participants (*“I think that’s more of a concern about missing*,* giving advice remotely and missing something. That’s more of a concern because people are more likely to come to harm because of that”* [S1])

### Stakeholder engagement

1. Facilitator: Stakeholders are generally willing to participate in remote postoperative monitoring, with online communication having been normalised over the pandemic.

Engagement of stakeholders is essential for the successful normalisation of an intervention. Online communication has been normalised over the pandemic for both patients and healthcare staff (*“we are so used to it and it is so handy and quick and takes literally no effort. We are all getting used to it*,* it is the norm for us lot.”* [P1]; *“there’s far more obviously since COVID I don’t think [telemedicine] really much of a thing before the pandemic…I think we’ve all got more comfortable with doing it whether we like it or not because it’s just the way of consulting now.”* [GP4]). Furthermore, data security was less of a concern for patients in this context - there was trust in the information governance around electronic health records, and data on postoperative recovery were not considered highly sensitive (*“Obviously my pictures were of my stomach with no face*,* so again that probably makes it a bit easier*,* even if you had a [data] leak”* [P6]).

2. Overall mixed facilitator and barrier: Patients are motivated to participate and generally feel comfortable with the tasks required, although some may struggle without additional training or support at home.

Patients highlighted the desire to remain well after surgery as a clear motivating factor to adopt the intervention, and also that postoperative patients are often recuperating at home and have time to engage (*“I was quite happy to do it*,* because obviously*,* I want to make sure my wounds are healing right”* [P14]; *“because you are doing absolutely nothing and you are recuperating it gives you something to focus on”* [P12]). However, they also reported that they needed to feel confident in their own capability for participation. In general, there was consensus that while patients felt to have the capacity to identify symptoms of complications like surgical-site infections, the majority did not necessarily have the insight into the significance (*“I think there’s always a bit of a niggling doubt in that have you missed something…you think it looks fine to me…you don’t always get a good view yourself.”* [P6]; *“what we view as a serious complication and what the patients view is a serious complication are miles apart”* [N2]). Within the context of the intervention, the patient-reported outcomes were considered to be understandable and low burden for patients to complete (*“It was quite straightforward*,* it was explained is there any redness? Is there anything coming out your wound? Whatever the question was*,* it was quite self-explanatory.”* [P10]; *“I think the number of questions were good. Anymore and it would be dragging on a bit…I probably would’ve stuck with it but I can’t speak for other people”* [P5]). However, there were concerns expressed regarding the digital literacy and support required for older patients to participate, with the process of photo capture being viewed as the greatest barrier to participation by patients (*“I couldn’t ever get the light right…I ended up*,* I got my husband to take the photographs. I could have taken them myself but the quality wasn’t wonderful”* [P9]).

3. Facilitator: Healthcare staff feel they can perform remote triage using information from postoperative monitoring, with a clear consensus that this only required sufficient experience and was not limited to only doctors.

For clinical benefits to be achieved, the information received as part of the intervention needs to allow healthcare staff to make clinical decisions. While there was a clear consensus that in-person assessment was the “gold standard” (*“There were very few instances where I felt it was enough; generally*,* I was itching to have them there face to face so I could examine them.”* [S6]), there was general agreement that patient-reported outcomes and photos were sufficient for initial assessment and triage of patients for in-person assessment (*“If they’re systemically well and you get a good picture with good quality of the wound and you’re able to say that looks fine and I think that would probably be fine and you would be comfortable to say no that’s all looking alright at the moment. I think the ones that look a bit off or the person isn’t feeling great*,* that would be much harder to make a judgement based on just remote stuff alone.”* [GP1])

It is also important to consider who will be conducting the review of these patient responses. There was recognition that both hospital and community teams have roles in the management of postoperative complications. Surgical teams were recognised as having that greater expertise in this, but also that often patients could be managed in the community (*“The wound can obviously be reviewed in the community but the operation was not done in the community so it is where the expertise is I guess.”* [S4]). Overall, there was acknowledgement that experience in managing postoperative complications was important for whoever had the role of reviewing (*“Whoever gets the job reviewing there has to be confidence in the decision-making…if you have a Nurse Practitioner who you have specifically trained to review and has the confidence to make the decisions that would be fine.”* [S4]), however that this may be speciality-dependent (*“you would think plastic [surgery] would have to have their own system because I wouldn’t be happy checking a wound for plastics*,* so it would have to be a sort of speciality specific thing…for orthopaedics*,* if you get it wrong and a patient ends up with osteomyelitis it’s a big problem.”* [N3]). Furthermore, there was clear consensus across hospital and community settings that nursing staff would be best placed to lead a remote postoperative monitoring service, so long as there was appropriate support from doctors (*“I think it could probably be done by someone who is sufficiently trained…you’d require a level of escalations*,* so you’d need another smaller tier above that.”* [S2]; *“But continual monitoring in primary care is not possible by a GP. It’s too expensive. It’s a bad use of resources*,* where other members of the team are cost less and are actually*,* probably be better at it.”* [GP5]).

### Organisational support

1. Overall mixed facilitator and barrier: Integration with existing health information infrastructures.

The health service also needs appropriate resources and support to sustainably deliver remote postoperative monitoring in the local context. There was agreement among stakeholders that the COVID-19 pandemic instigated positive change locally with nurse-led telemedicine services now normalised within local care pathways (“*We had great momentum for [telemedicine] during the main peaks of the pandemic*” [N2]; “*I think everyone knows that is the way everything is going and can’t see us having every patient in clinic again*” [P1]). However, particularly for digital health interventions, there is a strong need for sufficient technology and IT infrastructure. In this regard, staff highlighted the local context already has a primarily electronic health record with good integration of external platforms (*“I have worked with other systems elsewhere that are so much less clunky to navigate around*,* but the fact that you can get everything in one place with [electronic patient record] is great”* [S6]). However, it was noted there are separate electronic health records for community and hospital care with no capability for patient input of data. Furthermore, IT infrastructure was already viewed as an impediment to current practice (*“challenges are less about the electronic patient record itself but more about the platform on which it lives*,* so internet access*,* speed and IT accessibility*,* dropping signal when you go around the hospital.”* [S2];*“we are still having to battle over computers”* [S6]).

2. Overall mixed facilitator and barrier: It would be possible to integrate remote postoperative monitoring at present, however as the healthcare service is overstretched, this limits the scope without additional resources.

There also needs to be consideration of the implications on the workload for staff in the hospital and community. There is widespread concern about being able to maintain the existing workload, and so staff were apprehensive regarding change which could increase workload that was not matched by increased investment into staffing levels (*“If you were going to increase our workload we could just not manage it. At the moment with the state of play in General Practice then the answer would be zero*,* as we have no capacity for people who are desperate to see us.”* [GP2]; *“My only concern would be that you would have to have personnel to have the time in their job plans”* [S1]). Healthcare staff generally believed it would be possible to sustainably integrate a patient-led remote postoperative monitoring service into routine care. However, there was concern that a proactive (service-led) approach of routinely contacting the patient regarding their recovery would not be feasible (*“I do not think [patient-led approaches] will increase our workload*,* whereas having [regular prompts to complete then] someone is going to have to sit down and review many normal images.”* [S6]). However, this conflicts with patient preference, and may reduce the effectiveness of remote postoperative monitoring in practice (*“I think it would get very muddy if it was [patient-led]…you wouldn’t really be very sure is this just a niggle or I don’t want to bother them or I’ve forgotten all about it anyway*,* so i need to go to my GP.”* [P9]). A proposed compromise was to prioritise a service-led approach for only patients at high risk of postoperative complications to maximise benefits while minimising burden (*“I think it could be a mix of [patient-driven and service-driven contact]. So either patients who’ve had straightforward minor surgery are told here’s this*,* if you’ve got any problems with your wound*,* use this. But some of your more complex patients or your patients who are discharged with…you say could you do this day one*,* day three*,* day…”* [N1]).

## Discussion

### Summary

Without effective “normalisation” of complex interventions within local healthcare environments, successful and sustainable adoption cannot be achieved [9, 18]. This qualitative study provides a comprehensive understanding of a range of facilitators and barriers to digital health interventions in postoperative care across hospital and community settings, as well as direct comparison and contrast between the priorities of a range of different stakeholders. Telemedicine has been normalised during the pandemic across the NHS and other contexts [28–30], with participants interviewed able to recognise a purpose and clear benefit of digital remote postoperative monitoring service. However, this also creates a tension between patient needs and healthcare capabilities to deliver without additional resources, and so requires careful consideration of how these will be balanced to allow successful implementation within care.

### Does remote postoperative monitoring have a place within health services?

Even prior to the COVID-19 pandemic, the potential of DHIs to relieve the burden on the health system and improve delivery of care was recognised at governmental and organisational levels [28, 29]. In this study, there was consensus that remote postoperative monitoring had a clear application and clinical benefit to patients, staff, and health systems. This was primarily through early detection of postoperative complications, as well as improved utilisation and efficiency of healthcare services. These expectations align with published evidence on the perceived [31] and observed healthcare impact of remote postoperative monitoring [8, 14, 32, 33]. However, there have been longstanding concerns regarding the capability and willingness of both patients and healthcare staff to engage with remote postoperative monitoring [34, 35]. These appear to be less significant in practice [28, 29], with those interviewed perceiving this becoming normalised during the pandemic. In comparison to existing telephone-based services, participants interviewed perceived a distinct advantage to remote postoperative monitoring allowing visualisation and greater alignment of in-person review. However, this was not considered equivalent to in-person assessment, due to restrictions to important aspects of the diagnostic process such as communication, examination, and investigations. Despite a clear preference for in-person assessment, participants demonstrated a willingness to engage with remote postoperative monitoring as triage tool. However, there was also recognition that not all patients may have equal opportunity to participate in remote postoperative monitoring. This reflects wider concerns regarding a “*digital divide*” from differential smartphone ownership and digital literacy among already vulnerable patient groups [31, 36]. This will have diminishing significance over time as digital access and digital literacy continue to expand [37]. However, digital services will need to be adaptable to individual digital literacy and physical limitations with patients identified prior to discharge according to their digital literacy and potential support needs. In the meantime, patient groups who already face potential inequities in care can have these exacerbated unless traditional alternatives are continued, such as mail or telephone contact [38].

### How would health services deliver remote postoperative monitoring?

The clinical benefits of postoperative monitoring provide a strong driver for implementation. However, concerns remain around whether health systems can effectively deliver this potential [12, 29, 39, 40], with many being regarded as “*underfunded*,* underdoctored and overstretched*” [41]. Workforces under stress are less likely to engage in innovation, which may limit opportunities in practice [42, 43]. This was reflected by local healthcare staff who felt there was limited capacity for them to participate in additional activities. There is also a well-recognised lack of basic IT infrastructure and support within the UK health service [29, 39], reducing opportunities for DHIs to be integrated. Healthcare staff interviewed were negative regarding the existing infrastructure and support for digital transformation in the local context. Furthermore, the separation of electronic health records across community and hospital settings in the UK represents a well-recognised challenge to the provision of shared care [44]. Nevertheless, the local electronic health records were viewed positively in facilitating integration of external platforms which may form a bridge between care settings. Until these systemic barriers are addressed, a tension exists between patient and policy expectations regarding digital health, and the capacity of the healthcare service to deliver.

Overall, the burden on healthcare staff and the associated cost-effectiveness of the intervention will depend on several factors. Firstly, the volume and frequency of responses to review. There is agreement among participants in this study that it would not be feasible or an effective use of staff time to routinely review all patients for postoperative complications. Any form of formal remote postoperative monitoring was viewed as an improvement to routine care. However, there was disagreement between healthcare staff and patients, who often did not feel sufficiently informed to identify complications (desiring service-led follow-up), whereas healthcare staff generally felt patients should be empowered with this responsibility (desiring patient-led follow-up). Dynamics around the responsibility for identification of postoperative complications has been observed in similar studies investigating remote postoperative monitoring [11, 32, 45]. Prioritisation of service-led remote postoperative monitoring for those at the highest risk of complications (whether at a subgroup or individual patient level) may provide the optimal balance between stakeholder priorities. Secondly, the staff allocated to conduct remote triage. There was consensus among participants that staff involved require specialty-specific experience, but that shared care between hospital and community teams was needed. A nurse-led service was considered to be more easily normalised within the health system, so long as there was capacity for escalation to senior doctors. This is most similar to current practice in the local and other contexts [11] which feature nurse-led follow-up of high-risk patients. Thirdly, the diagnostic accuracy of remote triage. No method of screening has perfect sensitivity and specificity [26], and so there will always be a trade-off between reviewing all possible complications (avoiding false negatives) or only those with definite complications (avoiding false positives). An unacceptable combination risks undermining the potential benefits of remote postoperative monitoring, as well as the confidence and willingness to engage of both patients and healthcare staff [26]. This study reports the first evaluation of the “*minimally acceptable criteria*” of remote postoperative monitoring and the underlying rationale from participants (Fig. 3). Although there is consensus that the overall diagnostic accuracy should be maximised, there is a conflict between the prioritisation of specificity by patients and sensitivity by healthcare staff. Nevertheless, this depended on the “consequences” of a missed complication, with clear agreement that “serious” complications should almost always be identified.

Overall, sustainable integration of remote postoperative monitoring into existing care pathways will inevitably require restructuring of local health services, and appropriate allocation of resources and staff. These findings have been used to develop the first stakeholder-derived pathway for normalisation of remote postoperative monitoring service within routine care (Fig. 2). This is agnostic to the modalities used in remote postoperative monitoring, and so has the scope to be targeted towards complications of interest based on the clinical need and local capacity. Furthermore, the structure of the pathway empowers stakeholders to decide who should be prioritised for service-led contact (as opposed to patient-led contact) based on the risk of complications to minimise the volume of work; facilitates communication and monitoring across the hospital and community services; and recommends nurse-led review of online responses (with senior medical support) based on skillset and affordability. In the future, automated score or algorithm-based assessment of submissions may be able to mitigate the burden on staff, the cost required to deliver, and also allow large-scale, real-time triage to occur [46]. However, this also has independent barriers to implementation as the incorporation would require regulatory approval as medical devices [47]. Furthermore, it may disincentivise engagement due to the value that patients place on “speaking to a real expert” and wider apprehensions regarding the safety and trustworthiness within the healthcare context [48].

### Strengths and limitations

This qualitative study has several key strengths. Firstly, interviews were conducted in the context of the COVID-19 pandemic, a period of global and local digital transformation [49], and embedded within an ongoing local programme to implement remote postoperative monitoring [14, 33]. This provided interviewees with recent lived experience of digital health through participation in routine healthcare and/or the INROADE study. Secondly, this is one of the largest qualitative studies on the topic and involved a broad range of both patient and healthcare staff stakeholders. Thirdly, a hybrid theory-led analytic approach was adopted using the widely advocated implementation theory (NPT) combined with an inductive identification of sub-themes [18]. Together, these allowed a comprehensive identification and analysis of facilitators and barriers to remote postoperative monitoring, as well as direct comparison between the priorities of these different stakeholders. This has been used to develop the first published stakeholder-derived pathway for the normalisation of remote postoperative monitoring across primary and secondary care.

This study also has several limitations. Firstly, participants were from a single UK health board, and so facilitators and barriers identified will potentially reflect local healthcare delivery idiosyncrasies. However, these issues mirror those from other studies in the Global North [11, 13, 31, 50, 51]. While it is important to consider the local context and any unique aspects which may need to be accounted for, there can be broad similarities in how healthcare is delivered between different hospitals, health systems, and countries. It may be that only minor adaptations will be required when transporting an intervention to another context [52]. Secondly, interview-based research is inherently subject to self-selection bias, and so will lead to samples which may be more invested in the topic [53]. However, it is not possible, or even at times desirable, for qualitative studies to be representative of the wider population [54]. Instead, the selection was based on criterion-based purposeful sampling to provide a wide range of perspectives regarding the implementation across subgroups [16]. Finally, this study first and foremost focused on analysing surgical care pathways readiness for postoperative digital interventions. As such, the study specific focus meant that only stakeholders directly involved in these pathways, namely patients and healthcare staff were interviewed., The study did not directly incorporate the views from other stakeholders whose views would be important for broader and systemic implementation (e.g., policymakers, clinical managers, and information technicians). Therefore, there may be other barriers and facilitators to normalisation which have not been fully incorporated by user perspectives. While the importance of implementing DHIs like remote postoperative monitoring has been recognised at national levels [28, 29], financial, logistical, technical, and sociocultural factors at local levels which have been highlighted in this study will require engagement of all relevant stakeholders to support and sustain future successful implementations.

## Conclusion

The COVID-19 pandemic sparked unprecedented yet generally successful digital transformation across global health systems, with the use of telehealth within routine healthcare becoming accepted practice [34, 35]. This implementation was inherently reactive rather than strategic, with accelerating numbers of novel tools for remote postoperative monitoring continuing to be developed [8]. However, there remains no evidence-based recommendation on how these interventions should be implemented in practice. Furthermore, formal evaluation of interventions remains limited [8], and it remains unclear what aspects of these telemedicine services should be retained or redesigned moving forward. With the increased demand on surgical health services during post-pandemic recovery, novel approaches are needed to ensure stretched healthcare resources can be appropriately allocated. This work provides a clear understanding and cohesive path to normalisation of digital remote postoperative monitoring within routine healthcare. The benefits to stakeholders are clear, and if health systems seek to meet governmental policy and patient expectations, there needs to be greater organisational strategy and investment to ensure appropriate deployment and adoption in future perioperative care delivery.

## Electronic supplementary material

Below is the link to the electronic supplementary material.

Supplementary Material 1

## Data Availability

The transcripts generated and/or analysed during the current study are not publicly available to maintain confidentiality but are available from the corresponding author on reasonable request.

## Abbreviations

DHIs: Digital health interventions
ERAS: Enhanced Recovery After Surgery
GP: General practitioner
INROAD: ImplementatioN of Remote surgical wOund Assessment during the coviD-19
E: pandemic
IQR: Interquartile range
MAC: Minimally acceptable criteria
NPT: Normalisation process theory
PROMs: Patient-reported outcome measures
SWOT: Strengths, Weaknesses, Opportunities, Threats
SSI: Surgical-site infection
UK: United Kingdom
