# Supplementary material for: Implementation of digital remote postoperative monitoring in routine practice: a qualitative study of barriers and facilitators
Source: BMC Med Inform Decis Mak. 2024 Oct 21;24:307. doi: 10.1186/s12911-024-02670-5 (PMC11492749; doi:10.1186/s12911-024-02670-5)
Supplement: Supplementary file 1 — Supplementary Material 1 [file 12911_2024_2670_MOESM1_ESM.docx]

**Supplement**

**Supplementary Table 1: Normalisation process theory (NPT) domains and subdomains conceptualised for remote wound surveillance**

| **Domain** | **Sub-domain** | **Conceptualisation for remote postoperative wound surveillance** |
| --- | --- | --- |
| **Coherence** | 1. Differentiation | *Is remote wound surveillance distinct from routine postoperative care?* |
|  | 1. Individual specification | *Is it clear what tasks are involved for individual stakeholders in remote wound surveillance?* |
|  | 1. Communal specification | *Is there general agreement about the purpose of routine wound surveillance?* |
|  | 1. Internalisation | *Is it clear what the potential benefits and value of routine wound surveillance are?* |
| **Cognitive Participation** | 1. Enrolment | *Do stakeholders believe they are the correct people to be involved in remote wound surveillance?* |
|  | 1. Initiation | *Are stakeholders willing to participate and/or engage others in routine wound surveillance?* |
|  | 1. Activation | *Do stakeholders believe remote wound surveillance is an appropriate intervention for patients?* |
|  | 1. Legitimation | *What tasks and activities are required to integrate and sustain remote wound surveillance?* |
| **Collective Action** | 1. Interactional workability | *Does remote wound surveillance make it easier or harder for patients to be assessed for infection?* |
|  | 1. Skill set workability | *Do stakeholders have the correct skills and training to be involved in remote wound surveillance?* |
|  | 1. Relational integration | *Do stakeholders have confidence in remote wound surveillance compared to in-person assessment?* |
|  | 1. Contextual integration | *Is there sufficient organisation support and resources to allow implementation of remote wound surveillance?* |
| **Reflexive Monitoring** | 1. Systemisation | *Will stakeholders be able to judge the effectiveness of remote wound surveillance?* |
|  | 1. Individual / communal appraisal | *How will individuals / stakeholders overall judge the effectiveness of the remote wound surveillance?* |
|  | 1. Reconfiguration | *Are there suggestions from stakeholders that aim to modify and enhance the utility remote wound surveillance?* |

**Supplementary Table 2: Characteristics and outcomes of patients interviewed**

|  |  | **Patients Interviewed (n=14)** |
| --- | --- | --- |
| Age | Median (IQR) | 54.5 (48.8 to 57.2) |
| Sex | Male | 3 (21.4) |
|  | Female | 11 (78.6) |
| Ethnicity | White | 14 (100.0) |
|  | BAME | 0 (0.0) |
| Socioeconomic quintile | Median (IQR) | 4.5 (4.0 to 5.0) |
| Body Mass Index (BMI) | Not obese | 9 (64.3) |
|  | Obese | 5 (35.7) |
| Immunosuppression | No | 14 (100.0) |
|  | Yes | 0 (0.0) |
| Diabetes Mellitus | No | 13 (92.9) |
|  | Yes | 1 (7.1) |
| Operative approach | Minimally-invasive | 9 (64.3) |
|  | Open | 5 (35.7) |
| Operative complexity | Minor/Intermediate | 1 (7.1) |
|  | Major | 12 (85.7) |
|  | Complex Major | 1 (7.1) |
| Operative contamination | Clean-Contaminated | 14 (100.0) |
|  | Contaminated / Dirty | 0 (0.0) |
| Operative Urgency | Elective | 8 (57.1) |
|  | Emergency | 6 (42.9) |
| Clinical diagnosis of SSI (within 30-days) | No | 13 (92.9) |
|  | Yes | 1 (7.1) |

**Supplementary Figure 1: Schemata for exemplar smartphone-based intervention on remote postoperative wound monitoring.**


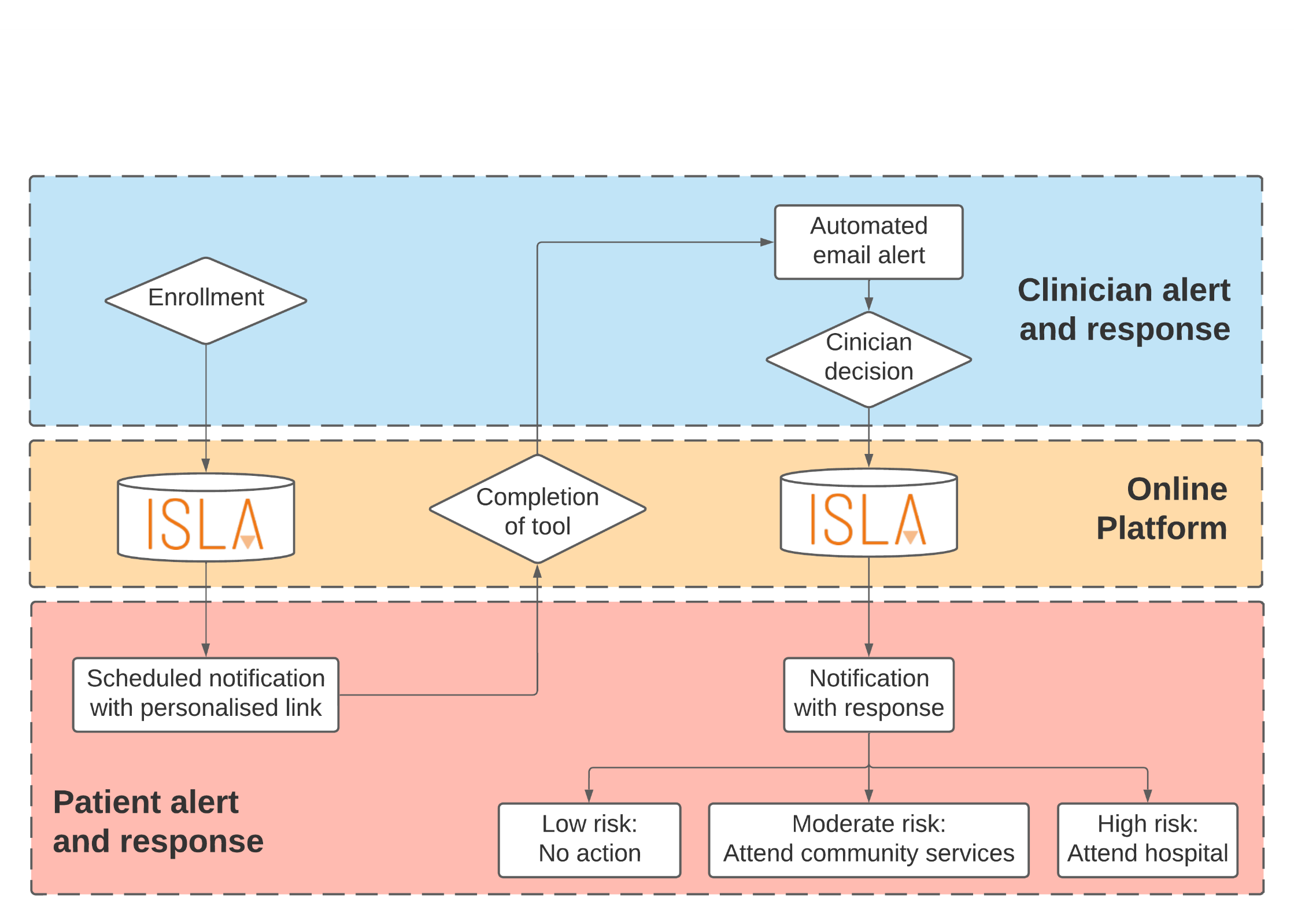


**Supplementary Figure 2: Patient stakeholder sentiment heatmap regarding implementation of remote postoperative monitoring**

**
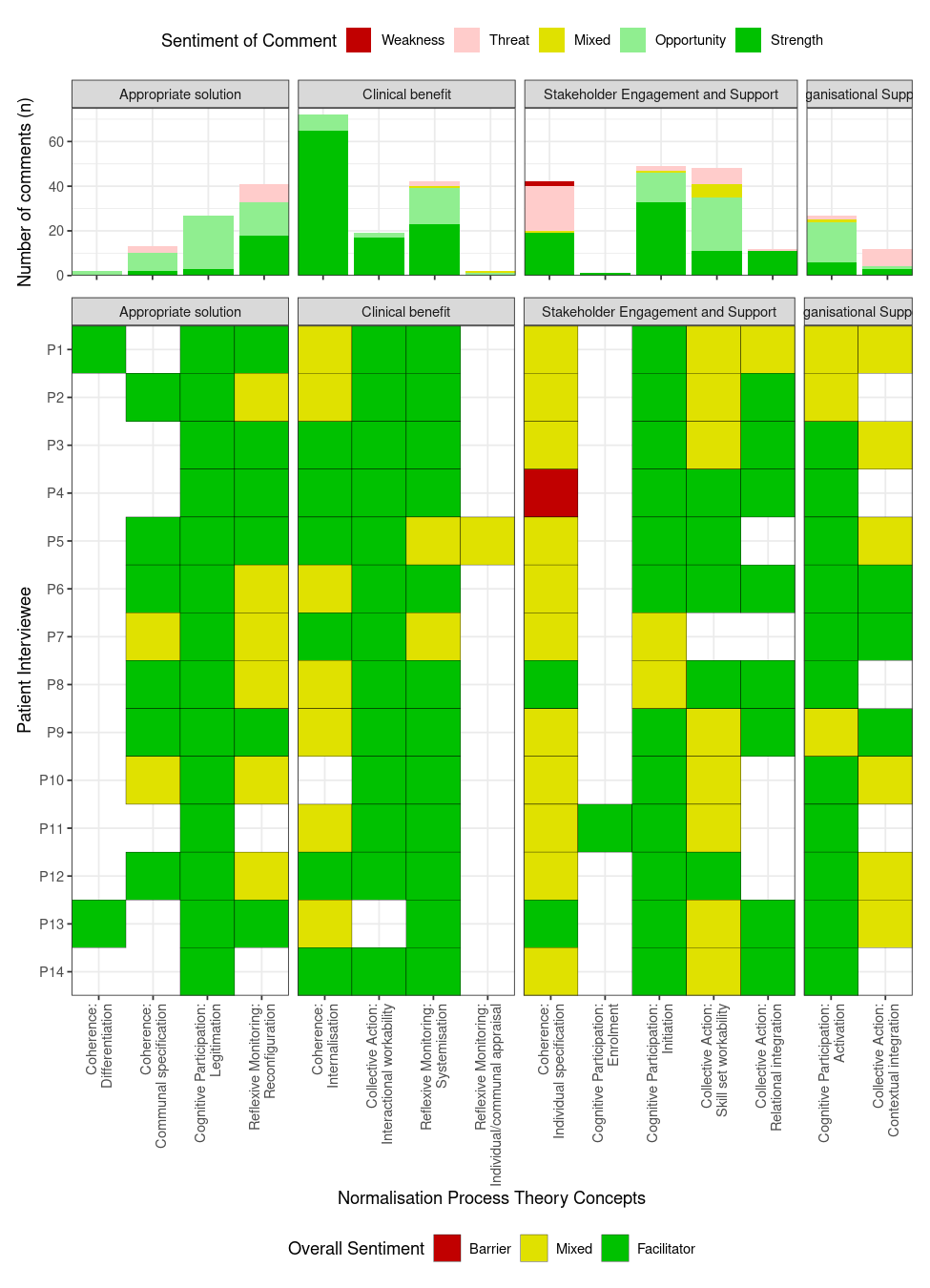
**

**Supplementary Figure 3: Healthcare staff stakeholder sentiment heatmap regarding implementation of remote postoperative monitoring**


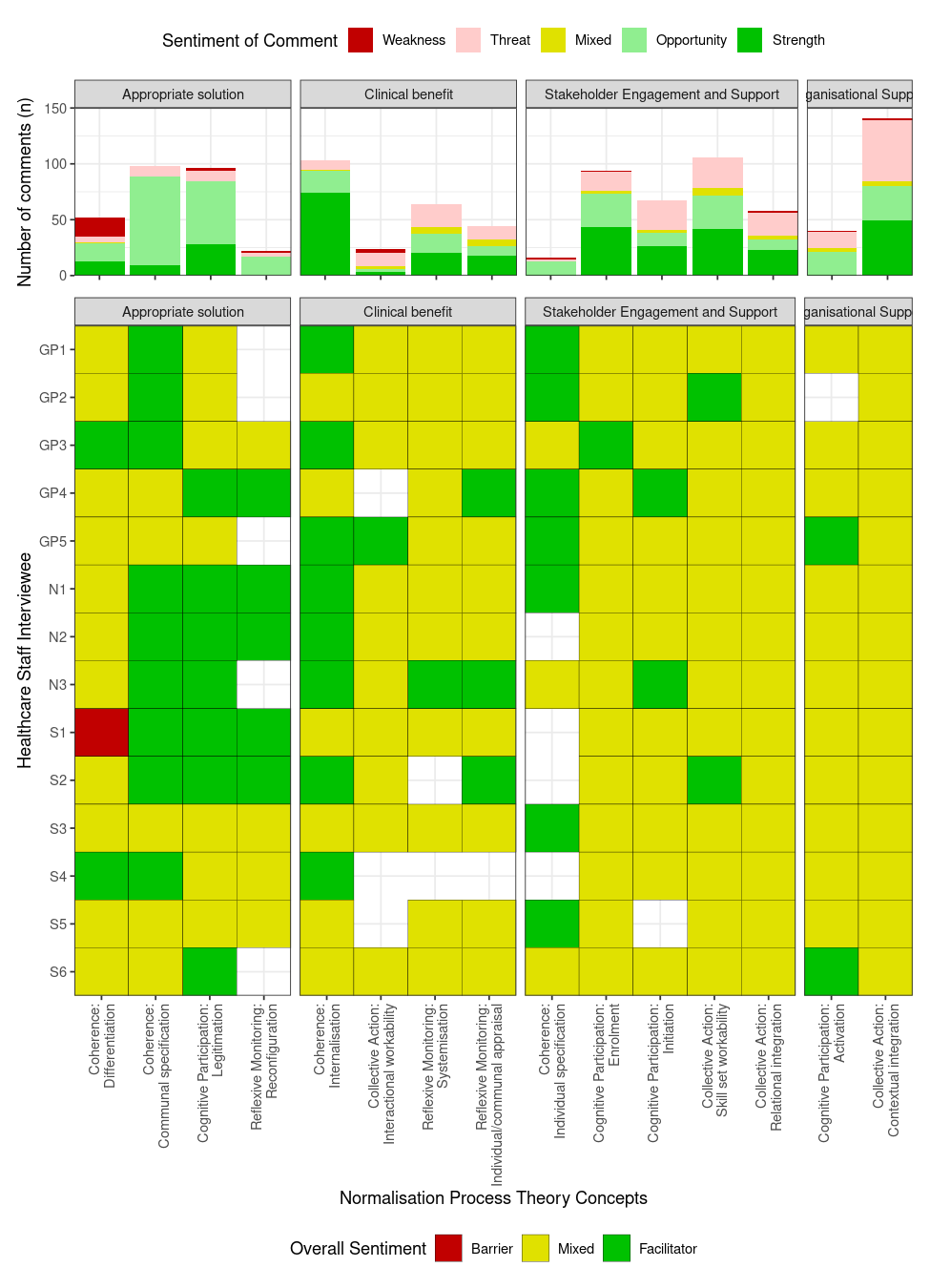


**Appendix A: Semi-structured interview guide for patients**

| **Experience of surgery and postoperative care** |
| --- |
| 1. Did you or the hospital staff have any concerns about your wound prior to discharge home? 2. How confident would you be in identifying problems with your wound? 3. What advice (written or spoken) were you given about what to do if you had any problems related to your surgery after you went home? 4. Did you have any concerns about how to get advice or care after you went home? |
| **Experience of the intervention** |
| 1. Did you or the hospital staff have any concerns about your wound prior to discharge home? 2. How confident would you be in identifying problems with your wound? 3. What advice (written or spoken) were you given about what to do if you had any problems related to your surgery after you went home? 4. Did you have any concerns about how to get advice or care after you went home? 5. How comfortable were you communicating with healthcare staff online? Any concerns? 6. How frequently would you like a mobile app to enquire about your recovery? 7. Prompt: Daily, every two days, twice per week, once per week? (if it was optional) 8. Would you prefer to have the ability to submit concerns about your wound when you wish, or would you prefer to be prompted? 9. Did you have any issues logging into the ISLA platform? Did the process feel secure to you? 10. Were there any questions asked on the form that you found confusing? How could this be improved? 11. Did you have any issues uploading photographs of your wound? How could this be improved? 12. How long did you have to wait before you got advice? Did you feel this was too long? 13. Did the advice provided seem correct? Did you follow this advice or did you seek a second opinion? How could the advice be improved? 14. Were you advised to see a doctor or nurse about your wound? If so, were you able to? If you were not able to, why was this? 15. If there were 100 people who get asked to go for a wound review (at their GP or hospital), what is the HIGHEST number of people you think should be asked to go but actually *don’t* have a wound infection? 16. If there were 100 people who are told their wound doesn’t need a review (at their GP or hospital), what is the HIGHEST number of missed infections you think would be ok? |

**Appendix B: Semi-structured interview guide for healthcare staff**

| **Interviewee Details** |
| --- |
| 1. What is your Job Title? Could you briefly describe your Role? 2. How long have you been in this position? |
| **Structure of the general surgical service** |
| 1. What parts of the electronic patient record do you access on a daily basis? 2. Are these fit for purpose / how could these be improved? 3. How often do postoperative complications arise in your experience? (in hospital and after discharge). What are the most frequent you see? 4. How are postoperative complications currently detected or come to the attention of the service / consultant? (in hospital and after discharge). 5. How are complications triaged / prioritised? 6. What do you consider serious postoperative complications? |
| **Current practice regarding SSI in postoperative care** |
| 1. If contacted regarding a patient in the community with a potential SSI, how are they cared for? (before covid and during covid) 2. On an average week, how many patients to you speak to (or review) with concerns regarding their surgical wound? 3. How reliable do you think diagnosing an SSI in-person is? 4. How important is it that surgical site infections are picked up early? |
| **Telemedicine applied to clinical practice** |
| 1. What areas of your practice or delivery of care could you see telemedicine affecting? 2. In your experience, do you think SSI can be reliably diagnosed using patient-reported symptoms? 3. In your experience, do you think SSI can be reliably diagnosed visually? 4. Would closer monitoring of patients in the community make you more comfortable sending patients home earlier? 5. How comfortable would you be communicating with your patients online? Any concerns? |
| **Design requirements for intervention** |
| 1. Should patients be able to contact any time with concerns with their wound or only at preset time periods? (e.g. on day 3, 7, and 15 after surgery). 2. Should all potential surgical-site infections in the community be reviewed (dealt with) by surgical teams? 3. If there were 100 people who get asked to go for a wound review (at their GP or hospital), what is the HIGHEST number of people you think should be asked to go but actually *don’t* have a wound infection? 4. If there were 100 people who are told their wound doesn’t need a review (at their GP or hospital), what is the HIGHEST number of missed infections you think would be ok? |
